# The role of serum Neutrophil Gelatinase-Associated Lipocalin (NGAL) in detecting acute kidney injury in preterm neonates exposed to nephrotoxic drugs

**DOI:** 10.1186/s12887-025-06432-8

**Published:** 2025-12-30

**Authors:** Marwa Eldegwi, Sally Hassan, Mo’men Saadoun, Hebatalla Ahmed, Heba Reyad, Ayat Elnahal

**Affiliations:** 1 Neonatology Unit, Department of Pediatrics, Faculty of Medicine, Kafr Elsheikh University, PO. 33516, Kafr Elsheikh, Egypt; 2 Medical Microbiology Department, Faculty of Medicine, Kafr El-Sheikh University, Kafr Elsheikh, Egypt; 3 Clinical Pathology Department, Faculty of Medicine, Kafr El-Sheikh University, Kafr Elsheikh, Egypt; 4 Community Medicine Department,, Faculty of Medicine, Kafr El-Sheikh University, Kafr Elsheikh, Egypt

**Keywords:** Acute kidney injury, NGAL, Neonates, Nephrotoxic drugs, Biomarker, NICU

## Abstract

Acute kidney injury (AKI) is a serious complication in neonates, especially among those exposed to nephrotoxic medications. Serum neutrophil gelatinase-associated lipocalin (NGAL) has emerged as a potential early biomarker for AKI, but its utility in neonates remains unclear. We aimed to assess the diagnostic performance of serum NGAL as an early predictor of AKI in preterm neonates receiving nephrotoxic drugs in the NICU. This prospective observational study included 70 preterm neonates admitted to the NICU at Kafr Elsheikh University Hospital between September 2023 and April 2024. Neonates receiving nephrotoxic drugs were enrolled, and serum NGAL and creatinine were measured on days 3 and 8 of admission. AKI was defined using modified KDIGO criteria. Comparative statistical analyses were conducted to assess NGAL's predictive value. AKI occurred in 30% of neonates. Serum creatinine and NGAL levels significantly increased after nephrotoxic drug exposure. However, no significant difference was observed between the AKI and non-AKI groups. While serum NGAL levels increased following nephrotoxic drug exposure, a single post-exposure measurement did not reliably predict AKI in preterm neonates. NGAL may have limited utility as a standalone biomarker for early AKI detection in this population.

## Introduction

Acute kidney injury (AKI) affects 18–70% of severely ill neonates and is often associated with poor outcomes for patients admitted to the neonatal intensive care unit (NICU). High-risk groups include patients with congenital heart disease, perinatal asphyxia, low birth weight or premature birth, necrotizing enterocolitis, and newborns who are given nephrotoxic drugs [1].

AKI is linked to a number of risk factors, including sepsis, toxicity, urinary obstruction, and hypoxia. Neutrophils and macrophages have a critical role in the inflammation associated with AKI. Neutrophils trigger inflammation by releasing cytokines, reactive oxygen species (ROS), proteases, and neutrophil extracellular traps into the renal tissue. Kidney macrophages express pattern recognition receptors (PRRs), such as Toll-like receptors (TLRs) and Nod-like receptors (NLRs), which recognize and activate pathogen-associated molecular patterns (PAMPs) and/or DAMPs. This mechanism activates intracellular signaling pathways like NF-κB and MAPK, resulting in the production of chemokines and cytokines. This promotes kidney inflammation by activating renal cells and recruiting circulating immune cells. Moreover, NK cells release IFN-γ, which triggers the production of inflammatory molecules and stimulates M1 macrophages in the kidneys. Th17 cells also generate IL-17A and boost the synthesis of chemokines, which encourage the recruitment of neutrophils and monocytes into the kidneys. Additionally, CD8 + T cells generate perforin and granzyme B, and they express Fas ligand, which is associated to cytotoxicity in kidney disease [2].

Preterm newborns are at risk for intraventricular hemorrhage (IVH) due to abrupt, substantial changes in blood pressure and electrolytes, so predicting the onset of AKI and altering fluid management promptly helps to reduce complications [3]. Blood-based renal markers provide objective evidence of AKI, e.g., serum creatinine (SCr) increases with decreasing kidney function. SCr is a simple and valuable technique for determining an adult population's glomerular filtration rate. Its application in neonates is, nevertheless, subject to numerous restrictions. The newborn's SCr during the first 72 h of life reflects maternal levels. In a neonate with normal renal function, the SCr will drop until it reaches a baseline, which may take up to six weeks for preterm babies and one to two weeks for full-term babies. Additionally, infants have little muscle mass, which is the basis for SCr levels [4]. Numerous studies have researched novel biomarkers to evaluate AKI events in early stages, improve clinical decisions, and allow for personalized treatment. The sensitivity and specificity of several glomerular and tubular biomarkers have been evaluated. The most promising seem to be neutrophil gelatinase-associated lipocalin (NGAL), interleukin (IL)−18, kidney injury molecule-1, β−2 microglobulin, and Cystatin-C [1, 4].

NGAL is a 26-kDa protein of the lipocalin family. It was initially found in activated neutrophils, NGAL regulates neutrophil development, adhesion, and phagocytosis and serves as a chemoattractant. Although the neutrophil is the primary source of NGAL production, NGAL is also produced by various cell types, including renal tubular epithelial cells, T lymphocytes and macrophages especially in response to ischemia, nephrotoxins, and systemic inflammation [5–9]. In AKI, pro-inflammatory cytokines such IL-1β and TNF-α increase the expression of NGAL in neutrophils, renal tubular epithelial cells, macrophages, and CD4⁺ T cells. NGAL can interact with Toll-like receptor pathways and play a role in immune signaling cascades that influence the renal inflammatory environment. It also has bacteriostatic properties, as it sequesters iron-laden siderophores, restricting bacterial access to iron. Furthermore, NGAL modifies immune cell functions by modifying neutrophil and macrophage recruitment, boosting regulatory T cell (Treg) activity, limiting oxidative stress, preventing apoptosis, and protecting epithelial integrity during AKI [10–13].

Epithelial damage and neoplastic diseases are associated with higher levels of NGAL. In AKI, NGAL is released by injured distal tubules. It is rapidly eliminated from circulation with a half-life of 10–20 min and reabsorbed by the proximal renal tubules, with levels rising within hours of renal insult; therefore, NGAL represents an early indicator of AKI [14, 15].

Additionally, NGAL is a biomarker for chronic kidney conditions, including immunoglobulin A (IgA) nephropathy, membranous, Pediatric LN, autosomal dominant polycystic kidney disease, and membranoproliferative glomerulonephritis [16]. Hence, our study aimed to assess whether serum NGAL can be used as a sensitive biomarker for early prediction of AKI in preterm neonates exposed to nephrotoxic drugs.

## Subjects and methods

### Study design

 This prospective observational study was conducted in the neonatal intensive care unit, Faculty of Medicine, Kafr Elsheikh University Hospital between September 2023, and April 2024.

### Sample size

The sample size was calculated using the Sample Size Online Calculator (https://sample-size.net/) [17]. The total minimum required sample size based on the previously mentioned criteria was 68 participants; we enrolled 70 participants to account for potential data loss. The calculation was based on the following assumptions:

1. Neutrophil gelatinase-associated lipocalin (NGAL) has an area under the ROC curve (AUC) of 0.776 for distinguishing patients with acute kidney injury (AKI).
2. Type I error (α) of 5% (corresponding to a 95% confidence level).
3. Statistical power of 80% (β = 0.20).

### Ethical approval

This study was approved by the Institutional review board of Kafr Elsheikh University (approval number: KFSIRB 200–143). The study details and procedures were explained to either parent of the study participants, and informed consent was obtained before enrollment. The procedures used in this study adhere to the tenets of the Declaration of Helsinki.

### Study participants

 The study included 70 preterm neonates who were receiving one or more potentially nephrotoxic medications—such as gentamicin, vancomycin, furosemide, ibuprofen, or acetaminophen—as part of their routine clinical care. Neonates with significant congenital anomalies or pre-existing renal conditions were excluded from participation.

### Data collection

Demographic details (gestational age, birth weight, length, post-natal age, gender, diagnoses); Appearance, Pulse, Grimace, Activity, and Respiration (APGAR) scores; drug-related details (name, dose, frequency, duration); laboratory parameters (serum creatinine, urine output, Serum NGAL). Neonatal Kidney Disease Improving Global Outcomes (KDIGO) classification was used in defining Acute Kidney Injury as any of the following [18]:

- Increase in serum creatinine by 0.3 mg/dL or more within 48 h or
- Increase in serum creatinine to 1.5 times baseline or more within the last 7 days, or
- Urine output less than 0.5 mL/kg/h for 6 h.

### Measurement of serum NGAL

Venous blood samples were collected aseptically from each neonate into serum separator tubes on the third and eighth days of hospitalization. Following collection, the samples were centrifuged at 2000–3000 RPM for 20 min. The resulting serum was aliquoted into 0.5 ml portions and stored at −80°C until further analysis. Serum creatinine levels were measured using an enzymatic method based on the principles established by Fossati, Prencipe, and Berti. For the determination of serum neutrophil gelatinase-associated lipocalin (NGAL), a commercial ELISA kit (BT LAB, Bioassay Technology Laboratory) was used, following the manufacturer’s protocol. In summary, the ELISA plate wells, pre-coated with human NGAL antibodies, captured NGAL from the serum samples. A biotinylated NGAL antibody was then added to bind the captured NGAL, followed by Streptavidin-HRP, which binds to the biotinylated antibody. After incubation, excess Streptavidin-HRP was removed by washing. A substrate solution was added, leading to a color change proportional to NGAL concentration. The reaction was stopped with an acidic solution, and absorbance was read at 450 nm.

### Statistical analysis

The data were analyzed using the IBM SPSS Statistics software (version 22.0; IBM Corp., *Armonk, NY, USA*; https://www.ibm.com/products/spss-statistics) [19]. Kolmogorov's test tests the normality of quantitative data. Qualitative variables were prescribed using numbers and percentage; the Chi-square test was used for analysis, or Fisher's exact test and the Monte-Carlo exact test (if more than 20% of the expected cell value is less than 5). Numerical variables were expressed as median (IQR), and the Mann–Whitney U-test and Wilcoxon Signed Ranks test were used to compare groups. A Receiver Operating Characteristic (ROC) curve was generated to assess the performance of serum NGAL in predicting AKI. *P-value* (< 0.05) was adopted as the level of significance.

## Results

A total of 70 neonates were included in the study, 51.4% male and 48.6% female. The median gestational age was 29 weeks (IQR: 27–31 weeks), and the median birth weight was 1105 g (IQR: 850–1500 g). The majority of the neonates were delivered by cesarean Sect. (84.3%). Notably, 82.9% of the neonates were appropriate for gestational age, while 17.1% were small for gestational age. APGAR scores at 5 min were predominantly in the range of 8–9, with a median CRIB score of 5.5 (IQR: 4.0–8.0). Maternal conditions such as pre-eclampsia, toxemia (22.9%), and premature rupture of membranes (17.1%) were common among the mothers (Table 1).

**Table 1:** Baseline Characteristics of the studied cases

Studied variables		*N* = 70	%
Sex	Female	34	48.6%
	Male	36	51.4%
Gestational Age (weeks)		29.0 (27.0–31.0)	
Birthweight (grams)		1105.0 (850.0–1500.0)	
Type of Delivery	CS	59	84.3%
	SVD	11	15.7%
Appropriateness of Gestational Age	AGA	58	82.9%
	SGA	12	17.1%
APGAR (5 min)	6.0	10	14.3%
	7.0	11	15.7%
	8.0	32	45.7%
	9.0	15	21.4%
	10.0	2	2.9%
CRIB Score (Clinical Risk Index for Babies)		5.5(4.0–8.0)	
Presence of Maternal Diseases	Pre-Eclampsia Toxemia	16	22.9%
	HELLP Syndrome	1	1.4%
	Antepartum Hemorrhage	2	2.9%
	ROP	12	17.1%
	Hypertension	2	2.9%

-Values are presented as numbers & percentages or Median (IQR)

-*HELLP syndrome* Hemolysis, Elevated Liver enzymes, Low Platelet count (a severe form of pre-eclampsia), *CS* Cesarean Section, *SVD* Spontaneous Vaginal Delivery, *AGA* Appropriate for Gestational Age, *SGA* Small for gestational age, *ROP* Premature Rupture of Membranes

In Table 2, Administration of nephrotoxic drugs led to significant changes in several laboratory parameters. The pH level increased from a median of 7.2 (IQR: 7.2–7.3) to 7.3 (IQR: 7.2–7.4; *p* < *0.001*). PaCO2 decreased significantly from 40.0 (IQR: 33.7–54.7) to 37.0 mmHg (IQR: 31.0–44.7; *p* < *0.001*), and base excess (BE) improved slightly from −8.1 (IQR: −11.3 to −5.4) to −7.8 (IQR: −9.5 to −5.9; *p* = *0.006*). Creatinine levels significantly increased from 0.6 (IQR: 0.5–0.7) to 0.7 mg/dL (IQR: 0.5–0.8; *p* = *0.001*), indicating potential kidney dysfunction. Additionally, serum NGAL levels rose significantly from 64.7 (IQR: 46.3–73.0) to 67.1 ng/mL (IQR: 58.7–80.3; *p* = *0.006*).

**Table 2:** Comparative analysis of laboratory parameters before and after administration of nephrotoxic drugs

Investigation	Before (*n* = 70)	After (*n* = 70)	*P-value*
pH level	7.2(7.2–7.3)	7.3(7.2–7.4)	< 0.001*^W^
PaCO2	40.0(33.7–54.7)	37.0(31.0–44.7)	< 0.001*^W^
HCO3	18.6(15.2–20.0)	17.7(15.7–19.9)	0.693^W^
BE	−8.1(−11.3- −5.4)	−7.8(−9.5–5.9)	0.006*^W^
Creatinine (mg/dL)	0.6(0.5–0.7)	0.7(0.5–0.8)	0.001*^W^
Serum NGAL	64.7(46.3–73.0)	67.1(58.7–80.3)	0.006*^W^

-Values are presented as Median (IQR)

^*^Significant, ^W^Wilcoxon Signed Ranks Test

**Fig. 1:**
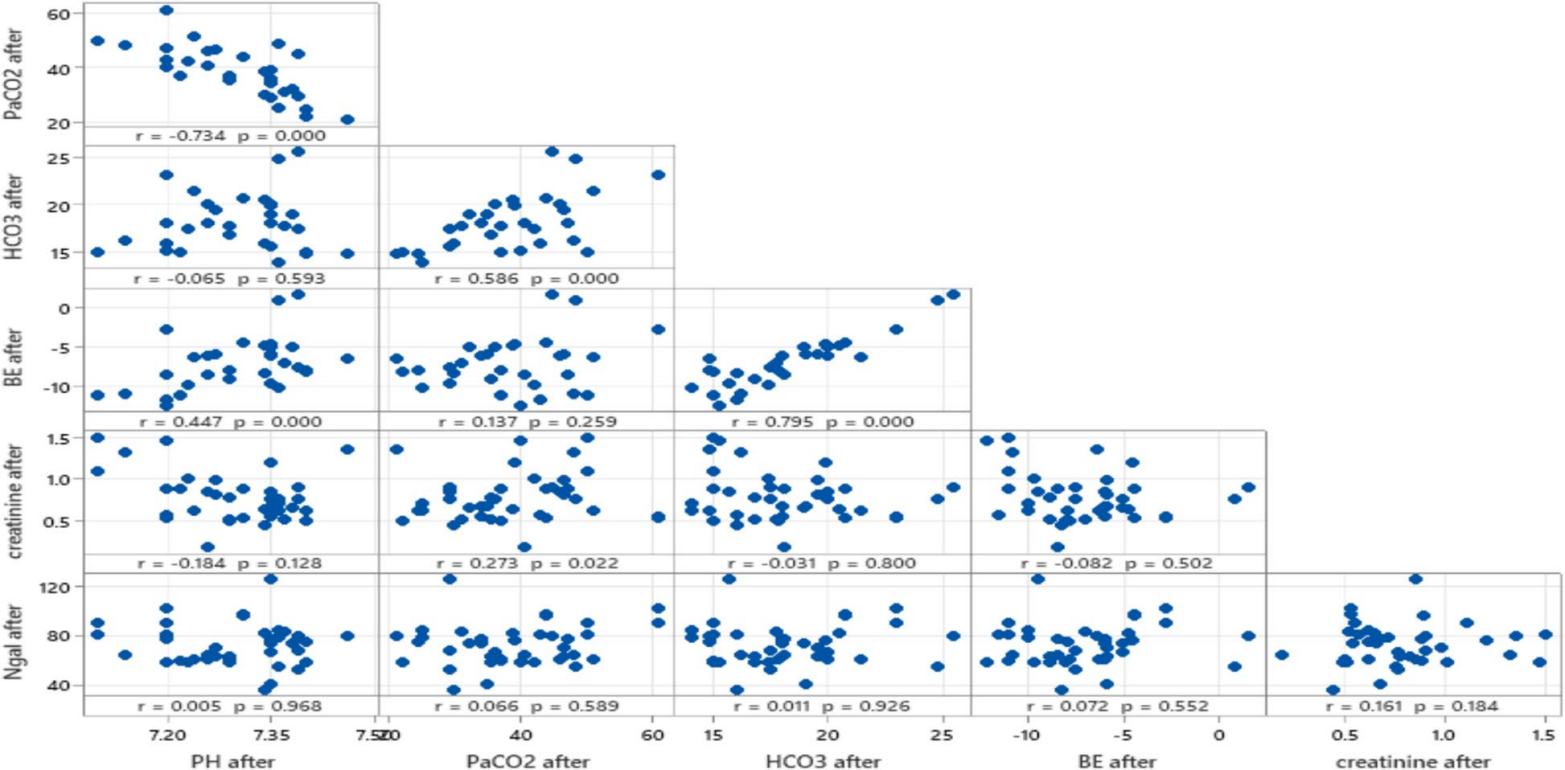
Correlation between NGAL and acid base parameters before exposure to nephrotoxic drugs

**Fig. 2:**
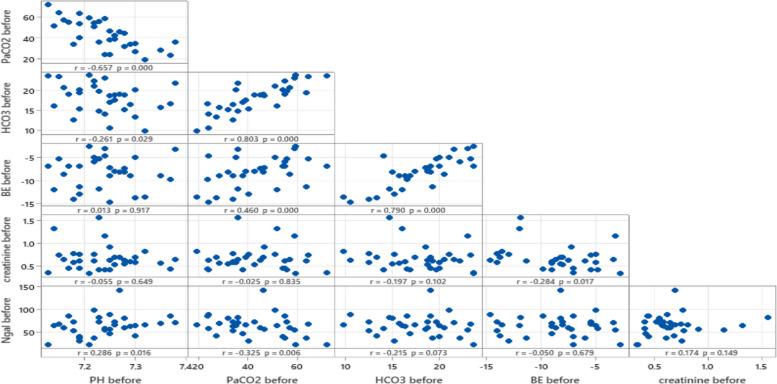
Correlation between NGAL and acid base parameters after exposure to nephrotoxic drugs

Figures 1 and 2 highlights that while NGAL shows limited correlation with traditional renal and acid–base parameters in this dataset, strong relationships exist among other metabolic and respiratory variables, such as BE, PaCO2, and HCO3. These findings reinforce NGAL's potential as a distinct early biomarker for kidney injury following nephrotoxic drug exposure.

Neonates with AKI (*n* = 21) had significantly higher creatinine levels (median: 0.9 mg/dL; IQR: 0.7–1.3) compared to those without AKI (median: 0.6 mg/dL; IQR: 0.5–0.8; *p* < *0.001*; Table 3). However, serum NGAL levels did not differ significantly between neonates with AKI (median: 60.9 ng/mL; IQR: 58.7–75.5) and those without AKI (median: 73.7 ng/mL; IQR: 61.2–81.9; *p* = *0.074*). Other parameters, such as pH, PaCO2, HCO3, and BE, showed no statistically significant differences between the two groups (Table 3).

**Table 3:** comparative analysis of laboratory parameters between neonates with and without Acute Kidney Injury (AKI)

Investigation	AKI (*n* = 21)	No-AKI (*n* = 49)	*P-value*
pH level	7.2(7.2–7.3)	7.3(7.2–7.3)	0.231^U^
PaCO2	40.0(37.0–48.0)	35.5(29.8–43.8)	0.091^U^
HCO3	17.5(15.2–19.9)	17.7(16.0–19.5)	0.918^U^
BE	−7.5(−10.8- −6)	−7.9(−8.9- −5.9)	0.739^U^
Creatinine (mg/dL)	0.9(0.7–1.3)	0.6(0.5–0.8)	< 0.001*^U^
Serum NGAL	60.9(58.7–75.5)	73.7(61.2–81.9)	0.074^U^

-Values are presented as Median (IQR)

^*^Significant, ^U^Mann-Whitney test

The incidence of oliguria was significantly higher in neonates with AKI (85.7%) compared to those without AKI (36.7%); *p* < *0.001* (Table 4). No significant differences were found in gestational age, birthweight, or the mode of delivery between the AKI and non-AKI groups. APGAR scores showed significant differences (*p* = *0.034*), with 19.0% of AKI neonates scoring six compared to 12.3% in the non-AKI group.

**Table 4:** Comparison of perinatal characteristics between neonates with and without Acute Kidney Injury (AKI)

Studied variables		AKI		No-AKI		*P-value*
		***N* = 21**	**%**	***N* = 49**	**%**	
Sex	Female	9	42.8%	25	51.0%	0.531^X2^
	Male	12	57.2%	24	49.0%	
Gestational Age in weeks		31.0(28.0–32.0)		29.0(27.0–31.0)		0.121^U^
Birthweight (grams)		1120(1000–1300)		1090(820–1500)		0.299^U^
Presence of oliguria		18	85.7%	18	36.7%	< 0.001*^X2^
Type of Delivery	CS	20	95.2%	39	66.1%	0.154^FE^
	SVD	1	4.8%	10	90.9%	
Appropriateness for Gestational Age	AGA	18	85.7%	40	81.6%	1.000^FE^
	SGA	3	14.3%	9	18.4%	
APGAR (5 min)	6.0	4	19.0%	6	12.3%	0.034*^MC^
	7.0	0	0.0%	11	22.4%	
	8.0	10	47.6%	22	44.9%	
	9.0	5	23.8%	10	20.4%	
	10.0	2	9.5%	0	0.0%	
CRIB Score (Clinical Risk Index for Babies)		6.0(4.0–8.0)		5.0(3.0–8.0)		0.565^U^
Presence of Maternal Diseases	Pre-Eclampsia Toxemia	2	9.5%	14	28.6%	0.196^FE^
	HELLP Syndrome	0	0.0%	1	2.0%	1.000^FE^
	Antepartum Hemorrhage	0	0.0%	2	4.0%	1.000^FE^
	ROP	2	9.5%	10	20.4%	0.325^FE^
	Hypertension	0	0.0%	2	4.0%	1.000^FE^

-Values are presented as numbers & percentages or Median (IQR)

-*HELLP syndrome* Hemolysis, Elevated Liver enzymes, Low Platelet count (a severe form of pre-eclampsia), *CS* Cesarean Section, *SVD* Spontaneous Vaginal Delivery, *AGA* Appropriate for Gestational Age, *SGA* Small for gestational age, *ROP* Premature Rupture of Membranes

^*^Significant, ^x2^chi-squared test, ^FE^Fisher’s exact test, ^MC^Monte Carlo exact test, ^U^Mann-Whitney test

**Fig. 3:**
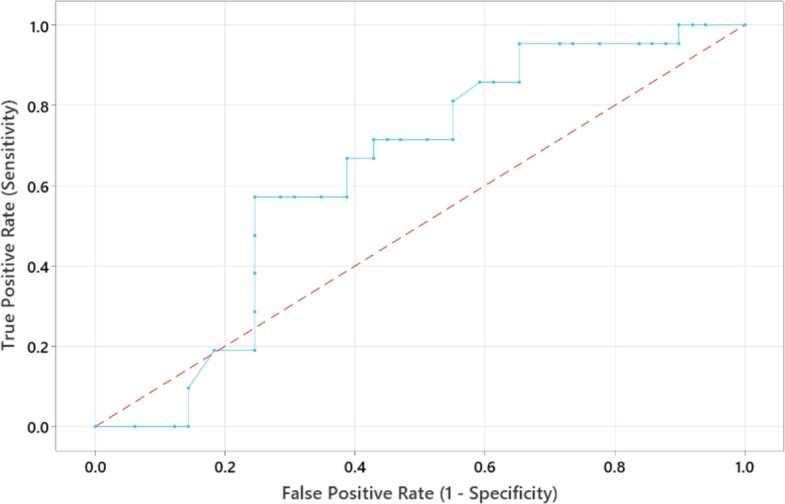
ROC curve analysis to assess the performance of serum NGAL in predicting AKI

None of the AKI neonates had an APGAR score of 7, while 22.4% of non-AKI neonates scored 7. Maternal conditions, including pre-eclampsia and toxemia, did not show statistically significant associations with the development of AKI. The ROC curve analysis demonstrated the performance of serum NGAL in predicting AKI. There was no significant difference between the AKI and non-AKI groups for the single fixed-day post-exposure serum NGAL measurement (AUC = 0.635, 95% CI: 0.493 to 0.768) (Fig. 3).

## Discussion

In the Neonatal Intensive Care Unit (NICU), the use of nephrotoxic drugs is often unavoidable, as non-nephrotoxic alternatives may not offer a more favorable balance between benefits and risks [20]. According to Steflik et al., during the first postnatal week, 75% of the newborns receive treatment with at least one nephrotoxic drug, such as aminoglycosides [21].

Nephrotoxic drugs represent a potentially modifiable cause of acute kidney injury (AKI). Hence, a proper and prompt diagnosis could prevent or lessen subclinical kidney damage and its consequences. Early diagnosis of AKI improves clinical decisions, enables personalized treatment, and eliminates the need for chronic follow-up [22]. Research into serum and urine biomarkers that are sensitive, specific, able to detect changes early after kidney injury, and simple to assess is ongoing. It is hypothesized that NGAL is a very sensitive and specific indicator of AKI, primarily of tubular cell damage, and serves as a putative biomarker for AKI [8]. Hence, this study aimed to determine the diagnostic performance of serum neutrophil gelatinase-associated lipocalin as an early predictor of acute kidney injury among newborns exposed to one or more potential nephrotoxic medications [8, 23].

A comparison of serum creatinine and NGAL levels before and after administration of nephrotoxic drugs revealed a statistically significant increase in creatinine levels from 0.6 to 0.7 mg/dL, and in serum NGAL from 64.7 to 67.1 ng/mL, suggesting drug-induced kidney injury with a potential impact on kidney function. Elevated Serum NGAL levels in AKI for a variety of reasons. The activation of neutrophils and monocytes during the acute phase of the reaction is one of the sources of NGAL in the serum. Furthermore, there is a marked increase in NGAL synthesis in the liver and lungs during AKI. Additionally, loss of renal function results in elevated serum concentrations [24, 25]. In the current study, 21 (30%) newborns were diagnosed with AKI according to the modified neonatal kidney disease improving global outcomes (mKDIGO) classification, which is the consensus definition utilized in research and clinically to diagnose and stage AKI [18]. In this context, Barhight et al. reported that nephrotoxic drugs represent a frequent cause of AKI in critically ill neonates with long-term complications, leading to potential chronic kidney disease. Moreover, AKI correlates with higher mortality rates, particularly in very low birth weight neonates. Age, comorbidities, drug dosage, duration of therapy, and concurrent drugs are some of the variables that affect the toxic effects of nephrotoxic medications [26, 27].

The detected incidence of nephrotoxic medications-associated AKI in this study (30%) is in line with the reported rates of AKI in the NICUs (18–70%). The observed wide range of AKI incidence in the NICU might be attributed to variations in AKI definitions and diagnostic methods, highlighting the need for a unified AKI definition and the variations in the studied populations [1].

The present study showed no significant difference in serum NGAL levels between AKI and non-AKI groups (AUC = 0.635, 95% CI: 0.493 to 0.768). These findings are consistent with the results of Kassem et al., who also reported that serum NGAL is not a specific biomarker for AKI prediction and loses its early predictive value (AUC = 0.56, 95% CI: 0.42–0.70) in critically ill children with sepsis-associated AKI [28]. In contrast, other studies have shown better NGAL performance in specific neonatal populations. For example, elevated serum NGAL concentrations were observed in preterm infants with respiratory distress syndrome who developed AKI [29], and Stoops et al. demonstrated that urinary NGAL levels increased following nephrotoxic drug exposure in the NICU, with more than a two-fold higher risk of AKI at concentrations ≥ 400 ng/mL (odds ratio = 2.76) [30].

Notably, NGAL is a part of innate immunity and is secreted by various tissues' epithelial cells and activated neutrophils. As a result, the rise in serum NGAL levels may not be unique to tubular injury but likely results from damage to other epithelial cells [8]. Because serum NGAL is influenced by some parameters, such as prematurity and inflammatory conditions, which limit the specificity of total NGAL measurement as a diagnostic of AKI, Suchojad et al. found that serum NGAL cannot be evaluated as a marker of AKI in preterm infants [31]. It has also been reported that the inflammatory status significantly impacts serum NGAL levels, with a strong correlation with inflammatory markers such as C-reactive protein [31, 32]. The neutrophil origin of non-monomeric NGAL, which influences the total NGAL levels, is another significant limitation of utilizing serum NGAL assays to indicate AKI [33]. However, the present study showed non-significant correlations between serum NGAL levels, blood pH, and PaCO₂ after administration of nephrotoxic drugs. This may reflect that changes in serum NGAL levels are more specifically related to kidney function rather than respiratory or metabolic disturbances.

Screening for nephrotoxic drug-induced AKI through systematic daily serum creatinine assessment decreased nephrotoxic medication exposure and associated AKI. However, daily venipuncture is invasive and associated with disposable and personnel healthcare costs [34, 35]. Introducing optional urine NGAL screening following exposure to high-risk nephrotoxic medications led to a 40% decrease in serum creatinine blood sampling and an increase in AKI screening adherence from 81 to 92% [36]. The possible role of serum NGAL in AKI screening programs following high-risk nephrotoxic medication exposure requires further research.

Most AKI patients included in this study were oliguric (85.7%), with a significant difference between AKI and non-AKI groups. In neonates, AKI might be oliguric or non-oliguric, with different frequencies in the studies [37]. Therefore, serum creatinine levels should be checked in patients with suspected AKI irrespective of the urine output. Furthermore, previous research has shown significant differences in the underlying risks and prognosis between oliguric and non-oliguric AKI in very preterm neonates. Oliguric AKI showed significantly higher mortality risks than non-oliguric AKI, regardless of serum creatinine levels and severity of AKI [38].

Interestingly, our study displayed a significant association between a low 5-min APGAR score and the development of AKI. The frequency of score 6 was significantly higher among AKI neonates than the non-AKI group (19.0% versus 12.3%, respectively). Also, none (0.0%) of the neonates in the AKI group showed a score of 7 compared to 11 (22.4%) in the non-AKI group. This finding agrees with the systematic review and meta-analysis that reported a low 5-min APGAR score as one of the significant risk factors for AKI among critically ill neonates [39]. Despite low gestational age and birthweight being widely recorded as risk factors for AKI in the NICU [37, 40], this study showed a homogenous distribution of the gestational age and birthweight among the AKI and non-AKI groups, with no significant differences between both groups. The development of AKI was also non-significantly associated with the sex of the newborns, mode of delivery, or maternal conditions such as pre-eclampsia or toxemia.

### Limitations

This single-center study included only 70 preterm neonates, which may restrict the generalizability of the findings. NGAL levels were not analyzed according to AKI severity, and only one post-exposure serum measurement was obtained. Although sampling occurred on days 3 and 8, these timepoints were insufficient to fully characterize NGAL kinetics after nephrotoxic exposure. In addition, systemic inflammation and sepsis may have influenced serum NGAL concentrations, potentially reducing biomarker specificity for renal injury.

## Conclusions and future directions

The results of this study indicate that exposure to one or more potential nephrotoxic drugs as part of standard treatment in the NICU is associated with increased serum NGAL levels. However, a single post-exposure serum NGAL measurement is not a reliable predictor of AKI development in preterm neonates. Larger multicenter prospective studies with serial serum and urine NGAL measurements are needed to better define the predictive value of NGAL after nephrotoxic exposure in neonates. Including term infants will clarify potential gestational age–related differences in NGAL performance and support the development of population-specific reference ranges.

## Data Availability

All data supporting this article will be made available by the corresponding author to any qualified researcher upon request.
